# Hearing in categories and speech perception at the “cocktail party”

**DOI:** 10.1371/journal.pone.0318600

**Published:** 2025-01-30

**Authors:** Gavin M. Bidelman, Fallon Bernard, Kimberly Skubic

**Affiliations:** 1 Department of Speech, Language and Hearing Sciences, Indiana University, Bloomington, Indiana, United States of America; 2 Program in Neuroscience, Indiana University, Bloomington, Indiana, United States of America; 3 Cognitive Science Program, Indiana University, Bloomington, Indiana, United States of America; 4 School of Communication Sciences & Disorders, University of Memphis, Memphis, Tennessee, United States of America; University of Southern Mississippi, UNITED STATES OF AMERICA

## Abstract

We aimed to test whether hearing speech in phonetic categories (as opposed to a continuous/gradient fashion) affords benefits to “cocktail party” speech perception. We measured speech perception performance (recognition, localization, and source monitoring) in a simulated 3D cocktail party environment. We manipulated task difficulty by varying the number of additional maskers presented at other spatial locations in the horizontal soundfield (1–4 talkers) and via forward vs. time-reversed maskers, the latter promoting a release from masking. In separate tasks, we measured isolated phoneme categorization using two-alternative forced choice (2AFC) and visual analog scaling (VAS) tasks designed to promote more/less categorical hearing and thus test putative links between categorization and real-world speech-in-noise skills. We first show cocktail party speech recognition accuracy and speed decline with additional competing talkers and amidst forward compared to reverse maskers. Dividing listeners into “discrete” vs. “continuous” categorizers based on their VAS labeling (i.e., whether responses were binary or continuous judgments), we then show the degree of release from masking experienced at the cocktail party is predicted by their degree of categoricity in phoneme labeling and not high-frequency audiometric thresholds; more discrete listeners make less effective use of time-reversal and show less release from masking than their gradient responding peers. Our results suggest a link between speech categorization skills and cocktail party processing, with a gradient (rather than discrete) listening strategy benefiting degraded speech perception. These findings suggest that less flexibility in binning sounds into categories may be one factor that contributes to figure-ground deficits.

## Introduction

Perceptual organization requires sensory phenomena be subject to invariance: features are mapped to common equivalencies by assigning similar objects to the same category membership [1]. Categories occur in all aspects of human cognition including face [2], color [3], and music [4–9] perception. But categories are particularly important in the context of spoken word recognition [10–13]. In speech perception, categories help bootstrap comprehension by generating perceptual constancy in the face of acoustic variability (e.g., talker variation, signal corruption) [14]. Thus, hearing in categories might help bolster speech-in-noise (SIN) skills by constraining and reducing perceptual variability in the speech signal.

Indeed, emerging evidence suggests that forming categories might benefit speech perception in noisy environments. In naturalistic soundscapes, the auditory system must extract target speech and simultaneously filter out extraneous sounds in what is described as the “cocktail-party problem” [15–17]. Theoretically, once equivalency between stimuli is formed, irrelevant variations among them can be deemphasized [1]. Based on this premise, we have theorized that hearing speech in a categorical mode (a more abstract level of coding) might help aid degraded speech perception since irrelevant variations in the physical surface features of the signal can be largely discarded in favor of retaining a more abstract, phonetic code for speech [18]. Supporting this notion, we have recently shown speech categories are surprisingly robust to acoustic interference, diminishing only at severe noise levels [i.e., negative signal-to-noise ratios (SNRs)] [18–21]. These behavioral results are bolstered by neuroimaging data which reveal the brain’s encoding of speech is not only enhanced for sounds carrying a clear phonetic identity compared to their phonetically ambiguous counterparts but that category members are actually more resistant to external acoustic noise [18, 22]. Similar parallels are found in the visual domain [23].

Further support for the link between categorical/discrete hearing modes of listening and SIN processing stems from studies in both highly skilled listeners and those with disorders. For example, musicians demonstrate improved figure-ground perception in a variety of SIN tasks [24–34], as well as better multi-talker cocktail party [35]. Musicians also show enhanced categorization for speech and musical sounds in the form of more discrete, binary labeling of tokens along graded continua [36–38]. On the contrary, several clinical populations involving auditory-based and learning disorders (e.g., dyslexia) can show weaker phoneme categorization [39–43] and poorer SIN processing [44–49] than their normally developing peers. The neural basis of acoustic-phonetic processing depends on a strong auditory-sensory memory interface [50–53] rather than higher-level cognitive faculties [e.g., attentional switching and IQ; 54]. Thus, the degree to which listeners show categorical (discrete) vs. gradient (non-categorical) perception could have ramifications for understanding clinical disorders that impair SIN processing. A failure to flexibly warp acoustic representations of the speech signal into well-formed, discrete categories could provide a linking hypothesis to describe individual differences in perceptual SIN skills among normal and clinical populations alike.

Conversely, an alternate view argues that gradient/continuous listening strategies might help facilitate SIN processing. Under this notion, maintaining sensitivity to within-category information (and even nuisance details of the noise itself) might allow more nimble perceptual readout of speech information [55, 56]. In other words, higher sensitivity to within-category information could offer more flexible processing, allowing listeners to “hedge” their bets in the face of ambiguity [55]. However, when tested empirically, gradient (non-categorical) perception is not always associated with speech-in-noise listening performance [55, 57]. This suggests that while listeners have simultaneous access to continuous, within-category cues [21, 58–61], they may not readily exploit them when parsing speech in ambiguous or degraded conditions [cf. 55]. On the contrary, both the construction of discrete perceptual objects and natural binning process of categorization might better enable category members to “pop out” among a noisy feature space, thereby facilitating SIN processing [e.g., 18, 62, 63]. Prior literature is thus equivocal on whether gradient or categorical modes of perception are more beneficial to SIN processing.

In this study, we examined SIN processing from the perspective of the “cocktail party” problem [16]. Such paradigms use more naturalistic acoustic environments that offer spatial cues for listeners to segregate target from competing speech information and engage binaural processing. Spatialization is an important acoustic cue listeners can exploit to parse multiple talkers and aid speech recognition in normal cocktail party scenarios [64]. This ecological component of normal auditory scene analysis is not testable using most clinical SIN tests conducted over headphones. Thus, our paradigm allowed us to (i) provide a comprehensive characterization of listeners’ “cocktail party” listening abilities and (ii) assess links between categorization and several domains of SIN processing including target speech recognition and localization accuracy, processing speed, and source monitoring abilities [35].

To this end, we measured speech-in-noise processing and phonetic categorization in young, normal hearing listeners to assess putative relations between these fundamental skills in speech perception. Because SIN perception might also relate to high-frequency hearing sensitivity even in “normal hearing” individuals [65, 66], we also measured extended high-frequency (EHF) audiometric thresholds as a control to rule out hearing sensitivity as a trivial factor that might account for putative categorization-SIN links. Noise-degraded speech perception abilities were assessed using standard clinical [i.e., QuickSIN; 67] and ecological SIN assays. For the latter, we used a simulated, multi-talker cocktail party task in a 3D auditory environment (anechoic chamber) to assess real-world SIN perception abilities that engage auditory segregation and cocktail party processes [35]. While some studies do show a connection between cognitive factors and laboratory-based speech-on-speech masking tasks [26], performance on our task is largely independent of cognitive factors including sustained attention, working memory, and IQ, suggesting it has high construct validity and is not easily explainable by mere cognitive differences between listeners [35]. Participants monitored target sentences [Coordinate Response Measure (CRM) corpus] [68] presented simultaneously with up to 4 additional talkers (other CRM sentences). Critically, we presented masking talkers in either a forward or time-reversed direction to induce more/less informational masking (IM). Informational masking (IM) is defined as the non-energetic aspect of masking interference that occurs for similar/confusable target and masker sounds (e.g., speech-on-speech). It typically represents additional central-cognitive aspects of figure-ground perception. In contrast, energetic masking (EM) is masking related to the physical interference of cochlear excitation patterns of the signal and masker and thus reflects more peripheral hearing function. Forward maskers were predicted to be more difficult since they are clearly recognized as speech carrying linguistic information and thus, should interfere with target recognition. The time-reversal in reversed maskers, on the other hand, largely destroys their lexical information and was expected to provide a “release from masking” [69]—making the task easier.

Categorization for labeling isolated acoustic-phonetic speech sounds was measured using two different continua [vowels vs. consonant vowels (CVs)] presented under different task structures (two alternative forced choice—2AFC vs. visual analog scale—VAS). These manipulations allowed us to assess categorization under stimulus and task conditions designed to promote discrete (2AFC) vs. gradient (VAS) hearing, respectively. CVs are perceived more categorically than vowels [11, 70, 71] and binary responding (2AFC) produces stronger categorical hearing during labeling than classifying the same speech sounds using a VAS scale [55]. Relevant to the current study, VAS categorization has been used to measure the degree of categoricity in a listener’s perception, since it allows for more graded judgments of the acoustic-phonetic space than a binary 2AFC task. Importantly, the VAS approach can identify listeners that respond in a discrete (categorical) vs. gradient (continuous) manner [55]. In this respect, we were particularly interested in VAS identification and 2AFC responses were measured largely as a baseline control. Based on prior work [18–21], we originally hypothesized that more categorical listeners (i.e., more binary responders) would show more successful QuickSIN and/or cocktail party speech perception. Alternatively, if a continuous listening strategy is more beneficial for SIN processing [55, 72, 73], more graded responders in VAS phoneme labeling should show improved SIN performance. To anticipate, our findings suggest a categorization-SIN link whereby more gradient (rather than discrete) categorization benefits degraded cocktail party speech perception.

## Materials and methods

### Participants

N = 21 young (age range: 22–37 years; 9 male, 12 female), normal-hearing adult participants were recruited for the study from the University of Memphis student body and surrounding community between 3/3/21 and 10/6/21. On average, they had 18 ± 1.1 years of education and were right-handed [72.6 ± 39.9% handedness laterality; 74]. All showed normal hearing sensitivity (puretone audiometric thresholds ≤25 dB HL, 250 to 20000 Hz; see **Fig 2**). We did not screen for subjective listening concerns. All reported no history of neurologic or psychiatric disorders. Non-native speakers perform worse on SIN tasks than their native-speaking peers [75, 76]. Thus, all participants were required to be native English speakers. The sample was largely “nonmusicians,” averaging 6.6 ± 6.2 years of formal music training [32, 77–79]. It should be noted that >10 years of music engagement is generally needed before observing musician-related benefits in SIN [32, 79] or cocktail party speech perception [35]. Indeed, participants’ years of musical training was not correlated with any of the dependent variables (all *p*s > 0.05). Each participant provided written informed consent in accordance with a protocol approved by the University of Memphis Institutional Review Board (#2370; approved 10/3/2012).

### Stimuli and task paradigms

#### Simulated cocktail party environment tasks

**Fig 1:**
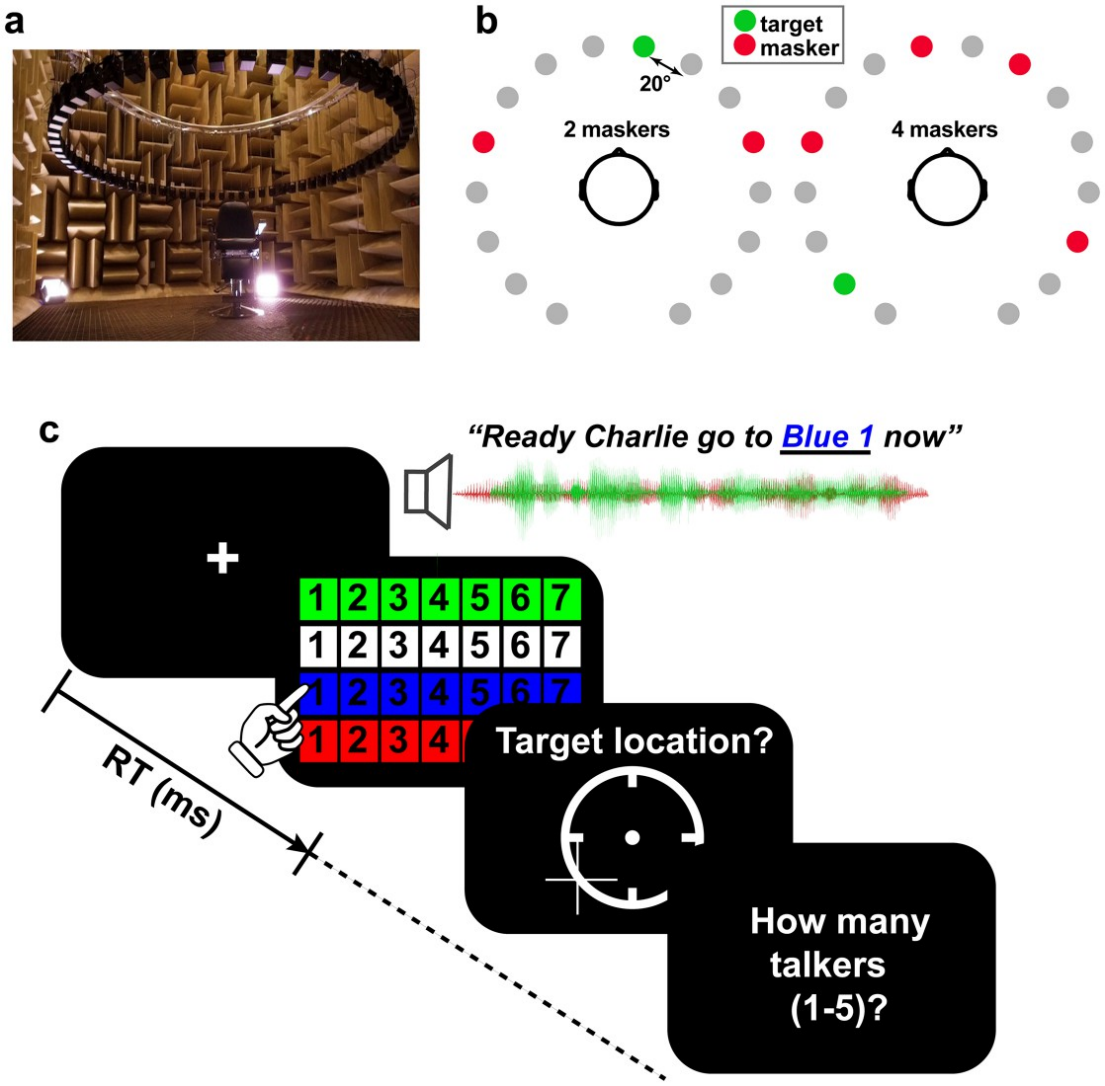
Cocktail party cocktail party task. (**a**) Participants were seated in the center of a 16-ch speaker array within an anechoic chamber. Speaker heights were positioned at ear level (~130 cm) during the task with a radial distance of 160 cm to the center of the head and speaker-to-speaker distance of ~20^0^. **(b)** Example stimulus presentation (2 and 4 masker talker conditions). Participants were asked to recall the color, number, and perceived location of target callsign sentences from the CRM corpus [68]. Target location was varied randomly from trial to trial and occurred simultaneously with between 0 and 4 concurrent talkers presented in either forward or time-reversed directions. (**c**) Example trial time course. After presentation of CRM sentences, listeners recalled the color-number combination of the target talker, its perceived location in the hemifield, and how many talkers they heard in the soundscape.

We measured naturalistic cocktail party listening skills via a sentence-on-sentence speech recognition task conducted in a 3D spatial soundfield [35]. Cocktail party speech perception was assessed in a simulated multi-talker cocktail party environment within the University of Memphis Anechoic Chamber (Fig 1a). The University of Memphis anechoic chamber is a room-within-a room design featuring a 24’ x 24’ x 24’ IAC chamber with floor/wall/ceiling Metadyne^®^ acoustic wedge coverage. The noise lock provides an STC 61 noise rating (low cutoff frequency = 100 Hz). A 36 channel Renkus-Heinz Model (CFX41) speaker array surrounds the seating location (16 were used in the experiment). Multichannel audio control is achieved by a TDT RX8 Multi-I/O Processor (Tucker Davis Technologies). Six Focusrite and Ashley Ne8250 amplifiers drive the speakers via a RedNet Dante MADI interface.

A 16-channel circular speaker array was positioned vertically 130 cm above the mesh floor of the anechoic chamber (approximately ear height). Subjects sat in the middle of the speaker array and were instructed to keep their head still during the task. Each speaker had a radial distance of 160 cm to the center of the head. Speaker-to-speaker distance was ~20 degrees.

We used Coordinate Response Measure (CRM) sentences [68] to measure speech recognition in a multi-talker sound mixture. CRM sentences contain a different target callsign (Charlie, Ringo, Laker, Hopper, Arrow, Tiger, Eagle, Baron), color (Blue, Red, White Green), and number (1–8) combination embedded in a carrier phrase (e.g., “Ready Charlie, go to blue three now”). The corpus contained all possible permutations of these callsign-color-number combinations spoken by eight different talkers (male and female). We used CRM sentences as they are not linguistically predictable to listeners and help avoid familiarity effects that might confound SIN performance [80–82]. They are also natural productions that offer a level of control (e.g., similar length, same sentence structure). Participants were cued to the target callsign before each block and were instructed to recall its color-number combination via a computer screen GUI as fast and accurately as possible (e.g., “b2” = blue-two; “r6” = red-six; Fig 1c). We logged both recognition accuracy and reaction times (RTs). RTs were clocked from the end of the stimulus presentation of the callsign (described below).

On each trial, listeners heard a mixture of sentences with one containing the target callsign and additional CRM sentence(s) that functioned as multi-talker masker(s). Three additional constraints were imposed on sentence selection to avoid unnecessary task confusion: (1) targets were always from the same talker and callsign (within a block); (2) maskers were absent of any callsign, color, and number used in the target phrase (i.e., the callsign’s information was unique among the speech mixture); (3) target and masker(s) were presented from unique spatial locations (i.e., different speakers). Male and female talkers were selected randomly. Thus, on average, targets and maskers were 50% male and 50% female. Presentation order and spatial location of the sentences in the 360-degree soundfield were otherwise selected randomly (Fig 1b).

We manipulated task difficulty by parametrically varying the number of additional maskers on a trial-by-trial basis (0 = target alone, 1, 2, 3, 4) presented at other spatial locations in the speaker array. All talker signals (i.e., target and individual maskers) were presented with an equivalent RMS level of 70 dB SPL (z-weighted, free field) [35], calibrated using a Larson–Davis sound level meter (Model LxT). Consequently, higher masker counts decreased the overall SNR making the task harder. We required participants to identify *both* the call color and number of the target callsign phrase to be considered a correct response (chance level = 3.13% = 1/32). It is possible for listeners to localize sound sources even if they cannot identify them [83]. Consequently, after recognition, we had participants indicate the perceived location (azimuth) of the target by clicking on a visual analogue of the speaker array displayed on the screen. Lastly, listeners indicated the number of total talkers they perceived in the soundfield to gauge source monitoring abilities [84]. An example trial time course is shown in Fig 1c.

This identical CRM task was run in two masking conditions: (i) forward and (ii) time-reversed maskers (random order). Forward maskers consisted of the CRM sentences unmanipulated. In the reverse condition, the masking talker sentences were time-reversed. These two conditions allowed us to assess listeners’ release from masking in acoustic interference while controlling for the SNR and long-term spectral characteristics of the maskers [85]. The difference between forward and reverse masker performance measures the release from masking [86], here resulting from the time reversal of the masker signal. There was a total of 64 trials per masker condition. Subjects were allowed a break halfway through the experiment to avoid fatigue.

#### Phoneme categorization

*Vowel and CV continua*. The vowel continuum was a synthetic 5-step vowel continuum spanning from “u” to “a” [18, 38, 71, 87]. Tokens were synthesized using a Klatt-based synthesizer coded in MATLAB [e.g., 88]. Each token was separated by equidistant steps acoustically based on first formant frequency (F1). Individual vowel tokens were 100 ms in duration including 5 ms of ramping. Each contained identical voice fundamental (F0), second (F2), and third formant (F3) frequencies (F0: 150, F2: 1090, and F3: 2350 Hz), chosen to roughly approximate productions from male speakers [89]. F1 was parameterized over five equal steps between 430 and 730 Hz such that the resultant stimulus set spanned a perceptual phonetic continuum from /u/ to /a/ [90].

The consonant vowel (CV) continuum consisted of a 5-step, stop-consonant /da/ to /ga/ sound gradient (varying in place of articulation) [e.g., 19, 71]. Original speech utterances were adopted from Nath and Beauchamp [91]. Individual CV tokens were 350 ms in duration including 5 ms of ramping. Stimulus morphing was achieved by altering the F2 formant region in a stepwise fashion using the STRAIGHT software package [92].

*2AFC vs*. *VAS categorization task*. Categorization for both continua was measured under two task paradigms: (i) 2 alternative-forced choice (2AFC) binary key press or (ii) mouse click on a visual analog scale (VAS) [54, 55, 93] (see **Fig 4**, ***insets***). 2AFC and VAS tasks were run in separate (randomized) blocks but used otherwise identical speech stimuli; only the task paradigm differed. The VAS paradigm required participants to click a point along a continuous visual scale with endpoints labeled “u”/”da” and “a”/”ga” to report their percept. The resolution of the VAS scale was limited only by the pixel width of the computer monitor (1920 pixels) and was effectively ~78 pixels/inch) given the width of the monitor (17.5”). Use of the entire analog scale was encouraged. Unless the participants had clarifying questions, no other instructions were provided [55].

Speech stimuli were delivered binaurally through Sennheiser HD 280 circumaural headphones. Listeners heard 15 trials of each individual speech token (i.e., 75 total = 15 trials*5 tokens) per 2AFC and VAS block. On each trial, they were asked to label the sound with a response (“u” or “a”; “da” or “ga”) as quickly and accurately as possible. Following listeners’ behavioral response, the interstimulus interval (ISI) was jittered randomly between 800 and 1000 ms (20 ms steps, uniform distribution) to avoid anticipation of subsequent stimuli. In total, there were four categorization conditions: /u/-/a/ and /da/-/ga/ continua presented in either a 2AFC or VAS paradigm.

#### QuickSIN

The QuickSIN [94] provided a normed test of SIN perceptual abilities. Participants heard six sentences embedded in four-talker noise babble, each containing five keywords. Sentences were presented at 70 dB HL. The signal-to-noise ratio (SNR) decreased parametrically in 5 dB steps from 25 dB SNR to 0 dB SNR. At each SNR, participants were instructed to repeat the sentence and correctly recalled keywords were logged. We computed their SNR loss by subtracting the number of recalled target words from 25.5 (i.e., SNR loss = 25.5-Total Correct). The QuickSIN was presented binaurally via Sennheiser HD 280 circumaural headphones using custom MATLAB scripts. Two lists were run and the second was used in subsequent analysis to avoid familiarization effects [32, 35].

#### Extended high-frequency (EHF) thresholds

In addition to standard pure-tone air-conduction audiometry, we measured hearing thresholds at EHFs of 9, 10, 12.5, 14, 16, 18, 20 kHz. EHFs were measured using circumaural headphones (Sennheiser HDA 200, Wedemark, Germany) specialized for high-frequency audiometry presented through a GSI AudioStar Pro audiometer (Grason-Stadler, Eden Prairie MN).

**Fig 2:**
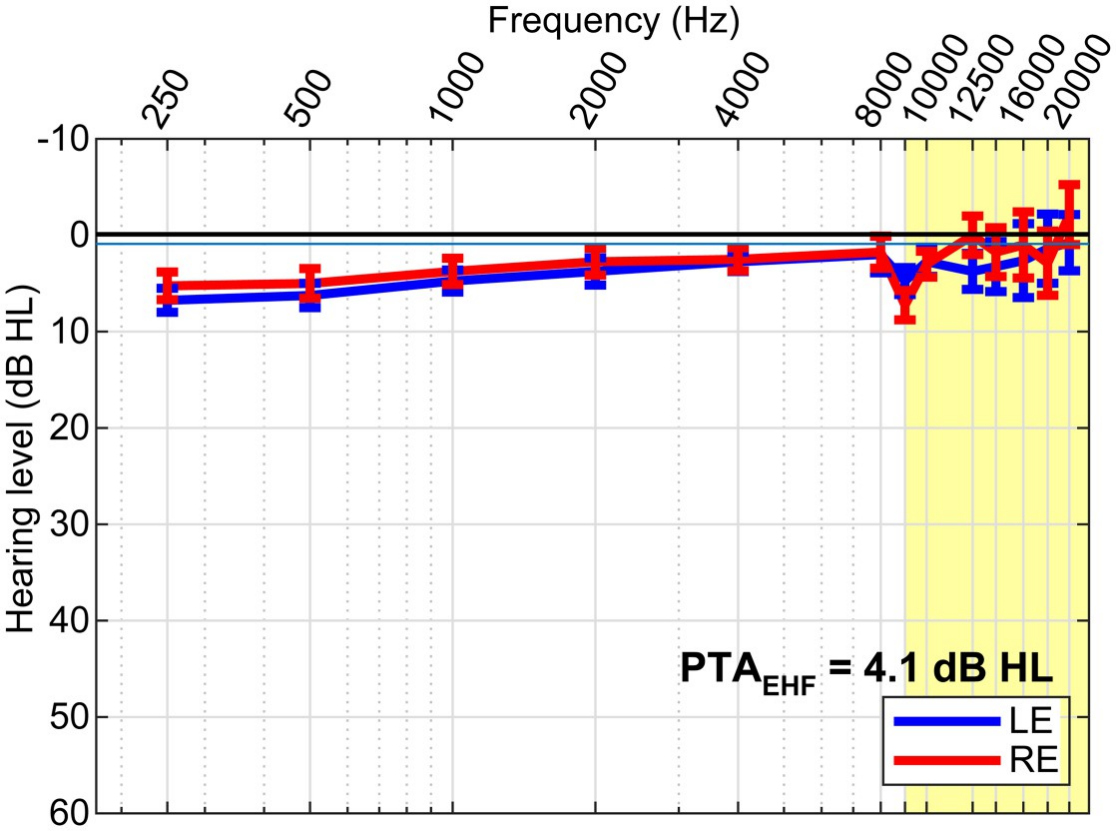
Extended high frequency (EHF) hearing thresholds. Audiograms for left (LE) and right (RE) ears. Pure-tone average (PTA) EHF thresholds in the normal and EHF (9–20 kHz; yellow highlight) frequency range were well within normal hearing limits. errorbars = ± 1 s.e.m.

History of middle ear infections might affect EHF thresholds [95]. Middle ear history in our listeners was unknown. However, the long-term effects of middle ear pathology (e.g., otitis media) typically shift EHF thresholds by 20 dB or more, whereas our subjects’ EHFs were near ~0 dB HL (Fig 2).

### Statistical analysis

Unless otherwise noted, we analyzed the dependent variables using mixed-model ANOVAs in R (version 4.2.2) [96] and the lme4 package [97] using maximum likelihood estimation. Speech cocktail party measures (%-accuracy, RTs, localization error, source monitoring) were analyzed with fixed effects of masker count (0–4) and masker direction (forward, reverse). Phoneme categorization measures (identification slope, RTs) were analyzed with fixed effects of task (2AFC, VAS), continuum (vowels, CVs), and—in the case of RTs—token (Tk1-5). Subjects served as a random effect. We computed the identification curve slopes for each condition as the rise/run change in %-labeling between tokens straddling the midpoint category boundary (i.e., vw2, vw4) [72]. Tukey-adjusted contrasts were used for multiple comparisons. %-correct data were RAU transformed prior to statistical treatment [98]. Slopes were transformed via sqrt[abs(*X*—mean(*X*))] to improve bimodality in the raw measure. Effect sizes are reported as partial eta squared ($\eta_{p}^{2}$) and degrees of freedom (*d*.*f*.) using Satterthwaite’s method. All tests were two-tailed.

## Results

### High-frequency thresholds

Grand average extended high-frequency (EHF) audiometric thresholds are shown for the left and right ear in Fig 2. EHFs in the 9–20 kHz frequency range were unremarkable (near 0 dB HL) for all listeners (average PTA_9-20kHz_ = 4.1 ± 10.5 dB HL).

### “Cocktail party” speech perception

**Fig 3:**
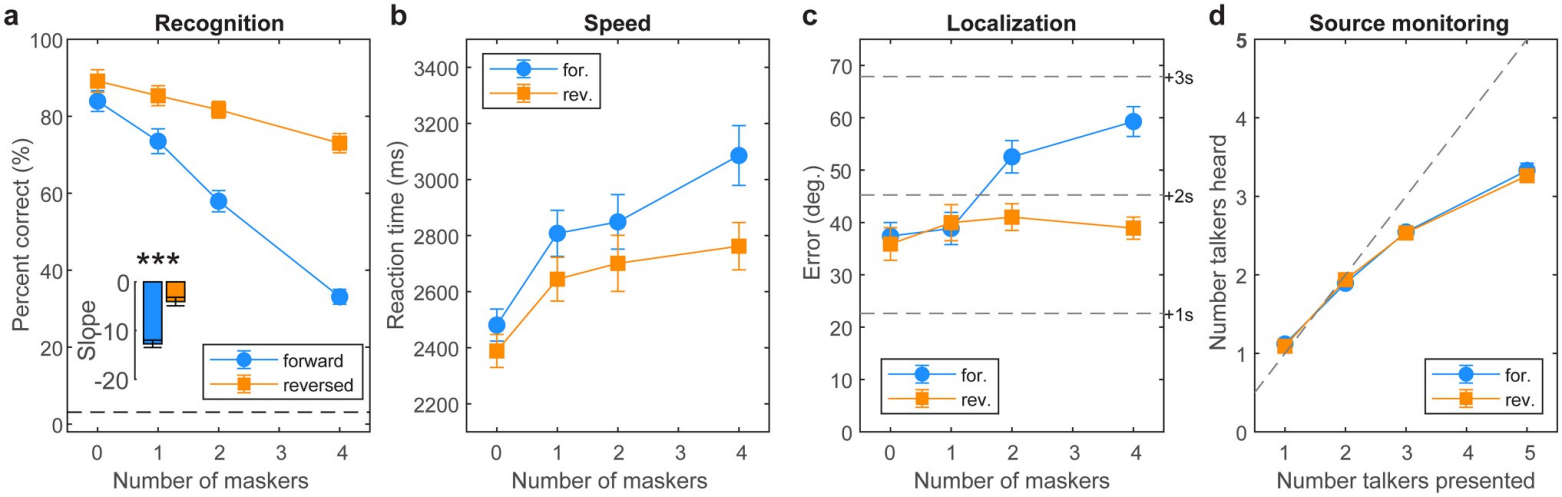
Cocktail party listening performance. (**a**) Speech recognition declines with increasing masker counts but is much poorer under informational/linguistic vs. purely energic masking (cf., forward vs. reverse masker directions). Dotted line = chance performance. (**b**) Owing to their added linguistic interference, forward maskers yield slower recognition speeds than reverse maskers. (**c**) Listeners localized targets within 2 speakers (40-60^O^ error) with better localization during purely energetic masking. (**d**) Source monitoring. Listeners saturate in source monitoring and only report hearing up to ~3 additional talkers despite up to 5 in the soundscape. errorbars = ± 1 s.e.m., ***p<0.0001.

Cocktail party performance measures (i.e., %-accuracy, RTs, localization error, source monitoring) are shown in Fig 3. Speech recognition expectedly declined from ceiling to near-floor performance with increasing masker counts from 0 (unmasked) to 4 maskers. Still, all listeners showed above-chance recognition even amidst 4 maskers (all *p*s< 0.0001; *t*-test against 3.13% chance). The main effects of masker count [*F*_*3*,*147*_ = 63.94, *p*<0.0001, $\eta_{p}^{2}=0.57$] and direction [*F*_*1*,*147*_ = 109.05, *p*<0.0001, $\eta_{p}^{2}=0.43$] on target speech recognition accuracy were significant. More critically, we found a masker direction x masker count interaction on recognition accuracy [*F*_*3*,*147*_ = 8.32, *p*<0.0001, $\eta_{p}^{2}=0.15$; Fig 3a]. The interaction was attributable to a stronger decline in speech recognition performance with increasing talkers amidst forward compared to reversed maskers (Fig 3a). This suggests target cocktail party was more challenging under conditions of forward compared to reverse masking loads.

For speed, we found main effects of masker count [*F*_*1*,*147*_ = 35.13, *p*<0.0001, $\eta_{p}^{2}=0.42$] and masker direction [*F*_*1*, *147*_ = 27.65, *p*<0.0001, $\eta_{p}^{2}=0.16$] on speech recognition RTs (Fig 3b) (interaction: *F*_*1*,*147*_ = 2.05, *p* = 0.11, $\eta_{p}^{2}=0.04$). These data reveal that decision speeds were predictably slower in more challenging multi-talker scenarios and with an increasing number of competing talkers.

Localization errors are shown in Fig 3c. Listeners localized targets within ~2–3 speakers (40-60^O^ error). Localization varied with both masker count and direction [interaction: *F*_*3*,*147*_ = 12.89, *p*<0.0001, $\eta_{p}^{2}=0.21$; main effect of masker count: *F*_*3*, *147*_ = 18.62, *p*<0.0001, $\eta_{p}^{2}=0.28$; main effect of masker direction: *F*_*1*, *147*_ = 34.72, *p*<0.0001$,\eta_{p}^{2}=0.19$]. Tukey contrasts show the interaction was attributable to masker-related differences at 2 and 4 masker counts. This suggests the influence of masker content (i.e., whether competing talkers were intelligible or not) was prominent only at higher talker counts.

Source monitoring is shown in Fig 3d. In general, listeners could distinguish how many talkers were in the soundscape with up to ~3 simultaneous voices. Performance plateaued thereafter suggesting a saturating effect in source monitoring performance. This was confirmed by a sole main effect of masker count [*F*_*3*,*147*_ = 636.73, *p*<0.0001, $\eta_{p}^{2}=0.93$]. The main effect of masker direction [*F*_*1*,*147*_ = 0.17, *p =* 0.68, $\eta_{p}^{2}<0.01$] and count x direction interaction effect were insignificant [*F*_*3*, *147*_ = 0.39, *p* = 0.76, $\eta_{p}^{2}<0.01$]. The lack of masker direction effect indicates source monitoring did not depend on masker intelligibility.

### Phoneme categorization

**Fig 4:**
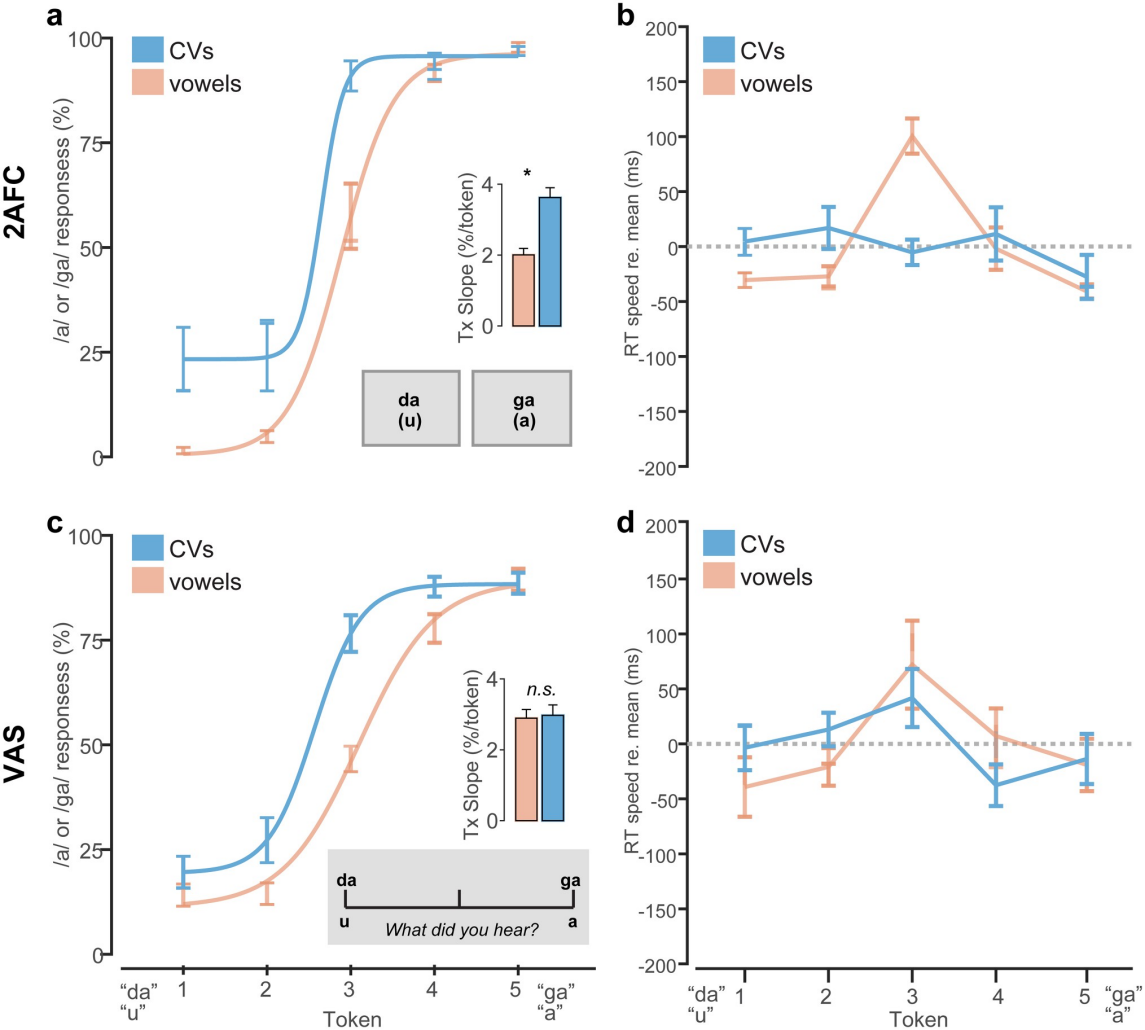
Stimulus- and task-dependent changes in the strength of perceptual categorization. Speech categorization and RT speeds under (**a-b**) 2AFC and (**c-d**) VAS labeling tasks. Note the sharper, more discrete categorization for CVs compared to vowels in the 2AFC (but not VAS) condition. RTs show the typical slowing near the perceptually ambiguous midpoint of the vowel (but not CV) continuum for both tasks. VAS responses were 750 ms slower than 2AFC across the board. RTs are plotted normalized to the global mean to highlight token- and stimulus-related changes. Identification slopes reflect sqrt[abs(*X*—mean(*X*))] transformed values. errorbars = ± 1 s.e.m., *p<0.05.

Phoneme categorization for CVs and vowels under the 2AFC vs. VAS task is shown in Fig 4. Identification slopes, reflecting the degree of categoricity in listener response pattern, were modulated by a main effect of stimulus [*F*_*1*,*84*_ = 11.59, *p* = 0.001, $\eta_{p}^{2}=0.12$] but not task [*F*_*1*,*84*_ = 0.23, *p* = 0.64, $\eta_{p}^{2}<0.01$]. More critically, we found an interaction between stimulus continuum and task [*F*_*1*,*84*_ = 9.58, *p* = 0.002, $\eta_{p}^{2}=0.10$]. Multiple comparisons revealed this interaction was due to steeper identification for CVs compared to vowels but only in the 2AFC task (Fig 4a). Slopes were invariant under VAS labeling (Fig 4c). These data support the notion that CVs are perceived more categorically than vowels [11, 70, 71]. However, the stimulus effect is not evident under tasks that promote continuous/gradient modes of listening, as in the VAS paradigm.

RT labeling speeds are shown in Fig 4b and 4d. RTs were ~750 ms later when categorizing speech sounds under VAS compared to 2AFC labeling [*F*_*1*,*394*.*3*_ = 1090.4, *p*<0.0001, $\eta_{p}^{2}=0.73$]. However, this effect is largely expected due to trivial differences in the nature of the motor response in the 2AFC vs. VAS tasks (i.e., keyboard vs. mouse). Consequently, for visualization purposes, we normalized RTs by subtracting the mean across tokens to highlight the *relative* changes in speed between continua and tokens [19]. An ANOVA conducted on raw RTs revealed main effects of token [*F*_*4*,*394*.*3*_ = 2.48, *p =* 0.043, $\eta_{p}^{2}=0.02$] and stimulus [*F*_*1*,*394*.*3*_ = 12.83, *p =* 0.0004, $\eta_{p}^{2}=0.03$]. All other 2- and 3-way interactions that included token, stimulus, and task were insignificant (all *p*s > 0.09). The stimulus effect was due to slightly faster (~70 ms) RTs for vowels compared to CVs. The token effect was attributable to the hallmark slowing (i.e., inverted-V pattern) in labeling speeds near the ambiguous midpoint of the continuum for vowels in both tasks [2AFC: *t*_*414*_ = 2.56, *p* = 0.011; VAS: *t*_*414*_ = 2.36, *p* = 0.0187] [58, 87, 88]. However, this slowing effect due to phonetic ambiguity was not observed for CVs under either task (*p*s > 0.29), consistent with prior work [71, 85]. These data support the notion that CVs are heard more categorically and with lesser phonetic ambiguity than vowels [11, 70, 71]. They also suggest the nature of the task changes categorization outcomes, with a 2AFC task structure producing more categorical/discrete hearing than a VAS task structure.

### Relations between listening categorization and cocktail party SIN perception

**Fig 5:**
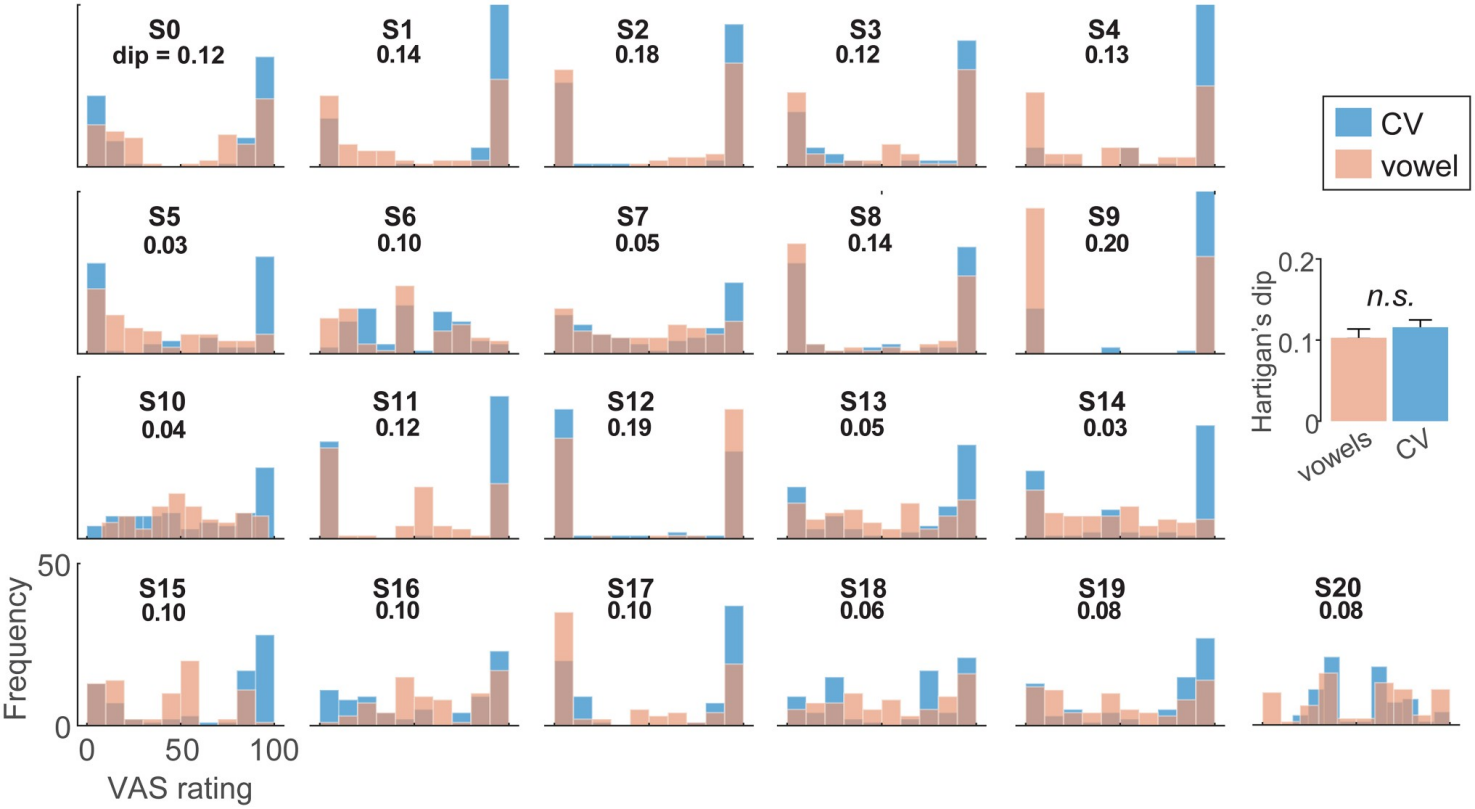
VAS ratings reveal stark individual differences in categorization and “continuous” vs. “categorial” listeners. Individual histograms show the distribution of each listener’s phonetic labeling for CV and vowel sounds. Discrete (categorical) listeners produce more binary categorization where responses lump near endpoint tokens (e.g., S2). In contrast, continuous (gradient) listeners tend to hear the continuum in a gradient fashion (e.g., S16). Inset values show Hartigan’s Dip statistic [99] score, quantifying the bimodality—and thus categoricity—of each distribution. Higher dip values = discrete categorization; low values = continuous categorization. (inset) Dip values are similar between CV and vowels suggesting it is a reliable measure of listener strategy that is independent of speech material. errorbars = ± 1 s.e.m.

Our phoneme labeling tasks were designed to promote more discrete (2AFC) vs. gradient (VAS) hearing. In particular, VAS ratings are thought to better isolate continuous vs. categorical modes of speech perception at the individual level [55, 72]. To quantify such individual differences in listening strategy, we divided our sample into “discrete” vs. “continuous” categorizers based on the distribution of their VAS labeling and Hartigan’s Dip statistic [99]. The Dip metric tests the intensity of bimodality of the data and thus whether labeling reports are bimodal (high dip score = categorical) or unimodal (low dip score = continuous) (Fig 5).

Being a discrete/continuous categorizer did not depend on speech content; Hartigan’s Dip statistic was similar between CVs and vowels [*t*_*20*_ = -1.15, *p* = 0.26] suggesting it was a reliable profile of individual listener strategy that is independent of speech material [see also, 72]. Given there were no stimulus-related differences in dip scores, we pooled CV and vowel VAS data for subsequent analyses. We then divided the sample into two groups based on whether an individual’s dip statistic computed from their VAS ratings showed significant (*p* < 0.01) evidence of bimodality. This resulted in two groups: “discrete” (n = 14) vs. “continuous” (n = 7) listeners.

**Fig 6:**
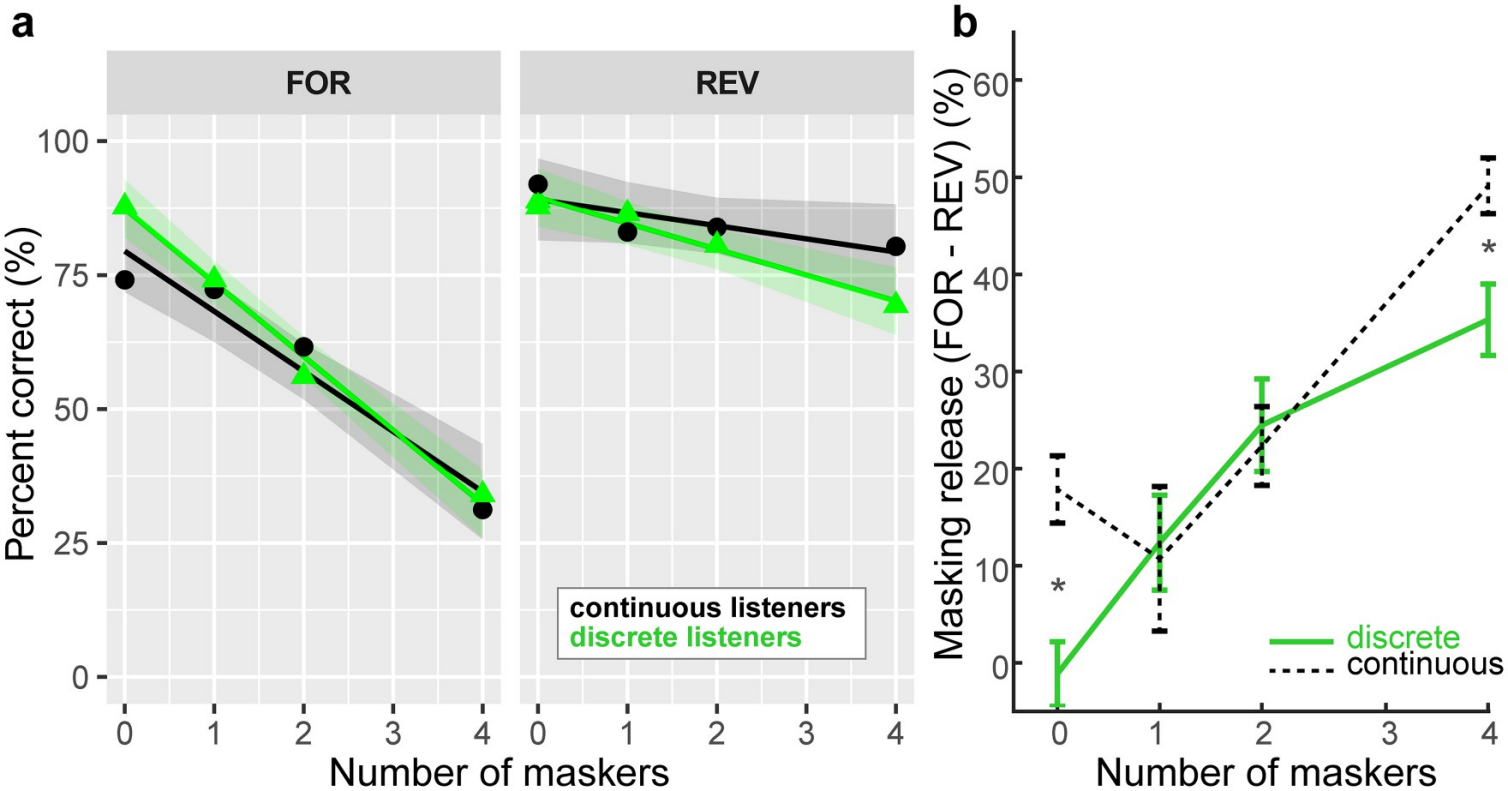
Gradient listeners are less susceptible to speech interference at the “cocktail party”. **(a)** Speech recognition performance in the cocktail party task for discrete and continuous listeners. Listener strategy was determined via Hartigan’s dip statistic [99] applied to VAS labeling (i.e., Fig 5) to identify individuals with bimodal (categorical) vs. unimodal (continuous) response distributions. Release from masking was measured as the difference in recognition performance between forward and reverse masker conditions at each masker count. (**b**) Discrete/categorical listeners show less masking release during speech cocktail party than their continuous listener peers. errorbars = ± 1 s.e.m.; shading = 95% CI; *p<0.05.

Fig 6a shows cocktail party speech recognition performance (as in Fig 3a) split by group. For each listener, we computed the degree of release from masking experienced in the speech cocktail party task, measured as the difference in recognition performance (raw %-correct scores) in the forward and reverse masker conditions at each masker count (Fig 6). The rationale behind this metric is that speech-on-speech masking in the forward talker condition contains additional linguistic interference due to the intelligibility of the masking talkers that further hinders figure-ground speech perception, whereas the reverse masking causes a “release from masking,” presumably due to a reduction in informational masking [30, 32]. Fig 6b shows masking release computed for “discrete” vs. “continuous” listeners. A 2-way ANOVA revealed main effects of masker count [*F*_*3*,*84*_ = 21.98, *p*<0.0001, $\eta_{p}^{2}=0.44$] and group [*F*_*1*,*84*_ = 4.90 *p* = 0.029, $\eta_{p}^{2}=0.06$] and masker x group interaction [*F*_*3*,*84*_ = 2.71, *p* = 0.050, $\eta_{p}^{2}=0.09$] on masking release (Fig 6b). The large masker effect was due to a steady and expected increase in masking release with increasing masker counts (i.e., larger performance improvement under REV vs. FOR maskers). The main effect of group indicates categorical/discrete listeners made less effective use of time-reversal and thus showed less release from masking than their gradient/continuous peers. Tukey post hoc comparisons indicated the masker x group interaction was partially attributable to more masking release in gradient listeners during the 0 masker (*t*_*92*.*8*_ = 2.76, *p* = 0.0069). Note that the difference in performance for the 0-masker condition is not really a “release from masking” (since there are no competing talkers) and probably reflects the fact that the target-alone condition was collected across different blocks. More importantly, the interaction was also attributable to more masking release in gradient listeners during the more difficult 4 masker condition (*t*_*92*.*8*_ = 2.00, *p* = 0.0479).

### Relations between EHFs and SIN

Correlations between QuickSIN and cocktail party measures were insignificant (all *p*s > 0.24), suggesting they tap different factors of auditory figure-ground processing. Similarly, QuickSIN was not related to any of the phoneme categorization measures (all *p*s > 0.14).

Despite all listeners having normal hearing, EHF thresholds did correlate with QuickSIN performance (Pearson’s *r* = 0.48, *p* = 0.0259). Slightly worse (though still within normal limits) high-frequency hearing sensitivity was associated with poorer (i.e., larger) QuickSIN scores. However, EHFs were not related to any measures of cocktail party performance (all *p*s > 0.05), indicating cocktail party perception was independent of high-frequency hearing. Similarly, EHFs were not related to the slope of listeners’ categorization functions (all *p*s > 0.50).

## Discussion

By measuring phoneme identification and degraded speech recognition in a multi-talker soundscape, we investigated links between two fundamental operations in speech processing: categorization and speech-in-noise (SIN) perception. Our findings suggest a more gradient listening strategy [72] promotes increased release from masking and thus aids “cocktail party” speech perception.

### Speech recognition at the cocktail party: Accuracy, speed, localization, and source monitoring

Our cocktail party speech task revealed that the ability to stream target speech amidst concurrent talkers depends critically on the linguistic nature of the maskers (i.e., whether or not they are interpreted as speech). Recognition accuracy and speed expectedly declined with increasing multi-talker interferers [35]. Poorer speech recognition with additional talkers is consistent with a reduction in spatial release from masking as more concurrent streams reduce the separability of the target in the soundfield [100]. More limited performance at higher masker counts also agrees with previous behavioral studies which show spatial release from masking is effectively limited to fewer than 6 sound sources [101].

Performance was also better overall during reversed compared to forward maskers. This effect was also anticipated and can be explained by the fact that forward maskers probably contain additional informational masking due to the linguistic information of speech-on-speech masking. In contrast, reverse maskers are easier to parse given they are not intelligible as speech, *per se*. Consequently, the forward talker condition containing speech-on-speech masking is more difficult given the added challenge of parsing multiple linguistic signals [30, 32]. The difference between forward and time-reversed conditions provides a measure of release from masking, which is typically attributed to central-cognitive aspects of figure-ground perception [102].

It is important to note there are many important cues that can provide release from masking (e.g., degree of spatial separation of target and masker, target-masker gender differences). For example, trials in which the target talker gender was different from other maskers would presumably result in much less IM than trials where the target and maskers were all the same gender. Maskers placed at further distances from the target location would presumably result in much less IM compared to closely spaced maskers. Trials with less IM would then have less masking to be released by other cues such as masker time reversal. These factors were randomized across trials in the present experiment. Consequently, a limitation of our study is that our task may over- or under-estimate the total possible masking (or IM) that could be “released.” Indeed, studies suggest that combination of release from masking cues (e.g., gender + time reversal, spatial separation + time reversal, gender + spatial separation) are not simply additive [103, 104]. As such, it is possible gradient listeners might experience even more advantages in SIN perception with additional release-from-masking cues.

In terms of localizing and monitoring talkers in the acoustic environment, we found listeners pinpointed targets within ~2–3 speakers (40-60^O^ error), consistent with our previous auditory cocktail party studies [35]. However, localization showed an interaction effect, suggesting the influence of masker content (i.e., whether competing talkers were intelligible or not) was more prominent only at higher talker counts. One explanation for this effect is that the localization task was delayed compared to recognition. There is evidence listeners can localize sound sources even if they cannot identify them [83]. Indeed, determining *where* a signal is emitted in the soundscape has a clear biological advantage over identifying *what* it is. Relatedly, our source monitoring results demonstrate that listeners are only able to identify the presence of ~3 talkers in the soundscape, despite more being present in the environment. This indicates a capacity limit in auditory cocktail party whereby listeners can only resolve up to ~3 distinct voices at any one time [present study; 84]. This finding is also consistent with channel capacity limits in auditory processing and notions that listeners cluster task-irrelevant sounds (e.g., background talkers) into a single stream to improve the perceptual segregation and identification of target information [105, 106].

### Categorization skills are related to SIN processing

VAS ratings of speech-sound continua allowed us to isolate continuous vs. categorical modes of speech perception and quantify individual differences in listening strategy based on phoneme labeling skills [55]. Applying this approach, we show listeners can be reliably pooled into “discrete” vs. “continuous” categorizers based on the distribution of their phoneme labeling. This division was not idiosyncratic to the specific speech content (i.e., whether listeners are identifying CVs or vowels), suggesting the behavioral profiles are a reliable index of individual listener strategy [see also 72]. Relevant to our original hypothesis of a categorization-SIN relation was listeners’ performance on the cocktail party tasks as a split of these functional differences in perceptual identification strategy.

Measuring the degree of release from masking experienced by listeners in speech cocktail party, we found SIN may be predicted by categoricity in hearing. However, the direction of the effect was opposite what we had originally anticipated. Interestingly, “discrete” listeners showed less release from masking and thus more speech-on-speech hindrance in performance than their “continuous” hearing peers. This group effect indicates that certain listeners who hear speech sounds in a more graded manner are less susceptible to interference at the cocktail party. This agrees with recent perceptual and electrophysiological studies that have linked gradient/continuous phonetic categorization to better speech in noise listening abilities [72, 73]. However, the current data disagree with studies examining musically trained listeners, who show both improved figure-ground perception in a variety of SIN tasks [24–35] and enhanced auditory categorization (i.e., more discrete identification) [36–38]. This leads us to infer that musicians’ putative SIN advantages reported in other studies are probably not due to categoricity in hearing and speech perception, but rather, broader central-cognitive factors (e.g., attention, working memory) [30, 32, 35].

That a gradient listening strategy is more beneficial to SIN processing is consistent with some prior work implying a benefit of continuous listening strategy [55, 107]. However, when put to empirical scrutiny, studies have failed to establish a consistent pattern between SIN performance and listening strategy [but see 72]. For example, word comprehension in noise for garden path and AzBio sentences does not correlate with listening strategy measured by VAS categorization [55, 57]. These findings, coupled with current results, suggest that while listeners can maintain access to continuous, within-category cues [21, 58–61], it is not always beneficial to parsing noise-degraded speech at the cocktail party. Instead, our data support the notion that hearing speech in a more graded mode aids degraded speech perception [cf. 18–21, 73]. Presumably, more graded/continuous perception allows listeners access to more detailed acoustic information in the signal, potentially allowing them to “hedge” their bets on what they are hearing in the face of noise and signal ambiguity [55, 72]. Further investigations into this result are warranted given the significant group difference found even for 0 maskers in the current data, and given that release from masking may have been over- or under-estimated in the current study.

On the other hand, some studies have suggested category-level cues provide easier readout to brain processing [18, 58, 90, 108, 109] and aid speech recognition in certain types of noise [18, 19, 22, 23]. Previous studies comparing phoneme categorization performed under clean vs. noise-degraded listening conditions reveal listeners easily label speech even at unfavorable SNRs [18, 19]. Categories might also aid the extraction of target speech percepts from interfering sound sources by reducing listening effort. This notion is supported by behavioral and physiological data [ERP: 18, pupillometry: 20]. Relatedly, perceptual warping effects in speech categorization [71, 110–113]—where tokens can be made to sound closer to distal prototypes in acoustic-phonetic space—are more prominent under noise relative to clean speech [21]. Indeed, in mousetracking studies on phonetic categorization, listeners take a more direct and faster motor path when classifying sounds amidst noise [21]. This could result from stronger perceptual attraction to category members [114], increased arousal/attention, or reduced decision ambiguity [115] supplied by the reductionist process of category mapping.

### Categorization is related to discreteness/gradiency rather than noise in perception

Categorization is typically quantified by the slope of listeners’ identification functions in a 2AFC task. However, shallower slopes in a 2AFC task may reflect perceptual gradiency and/or more internal noise in cue encoding. Both factors would tend to flatten a sigmoidal identification curve and thus are conflated in binary 2AFC tasks. Consequently, it has been argued that VAS labeling provides a purer measure of categorization discreteness/gradiency that is immune to the effects of sensory noise in behavior [55, 56]. The confounding of categoricity and sensory noise was also our primary motivation for using the Dip statistic [99] to define “categorical” vs. “continuous” listeners rather than identification slopes.

Still, to test the hypothesis that psychometric slopes reflect perceptual categoricity rather than internal decision noise, we estimated the noise in the VAS responses, measured as the *SD* in labeling reports across tokens [e.g., 55, 72]. Pooling across CV and vowel data, we found 2AFC slopes were not correlated with noise in the VAS task [*r =* 0.06, *p* = 0.79]. These findings thus do not support the assertion that a shallower slope (i.e., weaker categorization/more gradient listening) in a 2AFC task is due to increased internal sensory noise [cf. 55]. More critically, we found no correspondence between Dip statistic scores (bimodality of responses) and response noise [*r* = -0.06, *p* = 0.79]. Thus, our data suggest the slopes in 2AFC and VAS categorization tasks reflect the degree to which sounds are heard categorically rather than noisier responding, *per se*.

### Speech recognition in noise only partially relates to EHF thresholds

While our data suggest a perceptual link between categorization and SIN skills, it is worth acknowledging other factors that might drive listeners’ SIN abilities. For example, SIN performance has long been linked to higher-level cognitive skills—most notably, working memory [35, 55, 116, 117]. Prior studies have also suggested SIN perception in the form of cocktail party streaming is related to high-frequency hearing sensitivity, as measured via EHF thresholds, even in “normal hearing” individuals [65, 66]. This motivated the inclusion of EHF measures in the present study. In this vein, we observed a link between EHF audiometric thresholds and QuickSIN scores. Slightly worse (though still within normal limits) high-frequency hearing sensitivity was associated with poorer (i.e., larger) QuickSIN scores. However, we note EHF thresholds did not predict performance on the more complex cocktail party cocktail party task. The link between some SIN measures and EHFs is consistent with some [65, 66] though not all studies [cf. 118, 119]. Additional work is needed to understand putative relationships between high-frequency hearing and SIN abilities (even in normal hearing ears).

We also did not find a correlation between speech cocktail party measures and the QuickSIN performance. While at least a weak link between SIN measures might be expected *prima facie*, the lack of correspondence suggests these tasks tap different aspects of degraded speech perception. For example, the QuickSIN draws on figure-ground perceptual processing and is a threshold test, whereas our cocktail party tasks taps aspects of suprathreshold binaural hearing (release from masking) and attentional monitoring. The latter also features a more salient form of speech-on-speech interference and lexical competition that require listeners to resolve a level of informational masking that is not as prominent in the QuickSIN (speech on multi-talker babble). At the very least, the lack of correlations between cocktail party and both the (i) QuickSIN and (ii) EHFs we find in our data imply that standard clinical measures of SIN processing (e.g., QuickSIN) might be overly simplistic and fail to assess speech perception performance to the same degree as ecological cocktail party scenarios.

### Broader implications of a categorization-SIN link

Our findings suggest a link between two fundamental and arguably more rudimentary *perceptual* operations (categorization, figure-ground) that could explain broader individual differences in SIN skills among normal and clinical populations alike. For instance, the degree to which listeners show categorical vs. gradient perception might reflect the strength of phonological processing, which could have ramifications for understanding both theoretical accounts of speech perception and certain clinical disorders that impair sound-to-meaning mapping [e.g., dyslexia; 40, 120, 121]. It has even been suggested that deficits in speech categorization among certain developmental disorders might also be more prominent in noise [121]. Both categorization and speech-in-noise aspects of hearing show considerable *inter-*subject (but less *intra*-subject) variability [present study; 71, 73, 122–125]. Thus, it is tempting to infer that figure-ground deficits observed in some auditory and language-based learning disorders [44–49] result from a failure to flexibly warp category representations of the speech code. On one hand, graded/continuous perception might be advantageous for speech perception in noise since it would allow listeners access to all acoustic information in the signal, potentially allowing them to “hedge” their bets on what they are hearing in the face of ambiguity [55]. On the other hand, if a large portion of the perceptual space is corrupted by noise, hearing in discrete units might be preferable to allow category members to “pop out” among the noise and facilitate speech processing [18, 61, 62]. Our data here lead us to infer that the maintenance of detailed, graded auditory information is more beneficial to parsing speech in realistic cocktail party SIN scenarios and how well a listener can extract (or suppress) concurrent speech information. Nevertheless, future studies in clinical populations are needed to determine if SIN deficits commonly observed in clinical disorders truly result from deficits in sound-to-label mapping.

## Supporting information

S1 Data (ZIP)
